# Context‐Dependent Physiological Responses in *Scurria* Limpets Are Not Associated With Latitudinal Gradients or Biogeographic Breaks Across the South‐Eastern Pacific

**DOI:** 10.1002/ece3.72250

**Published:** 2025-10-10

**Authors:** Pablo Saenz‐Agudelo, Roberto F. Nespolo, Bernardo R. Broitman, Paz Caballero, Marco A. Lardies

**Affiliations:** ^1^ Instituto de Ciencias Ambientales y Evolutivas Universidad Austral de Chile Valdivia Chile; ^2^ ANID‐Millennium Science Initiative Nucleus (NUTME) Las Cruces Chile; ^3^ Biosecurity Group Cawthron Institute Nelson New Zealand; ^4^ Center of Applied Ecology and Sustainability (CAPES) Santiago Chile; ^5^ Millennium Nucleus of Patagonian Limit of Life (LiLi) Valdivia Chile; ^6^ Institute for Integrative Biology (iBio) Santiago Chile; ^7^ Departamento de Ciencias, Facultad de Artes Liberales Universidad Adolfo Ibañez Viña del Mar Chile; ^8^ Instituto Milenio en Socio‐Ecología Costera (SECOS) Santiago Chile; ^9^ Escuela de Ciencias del Mar Pontificia Universidad Católica de Valparaiso Valparaiso Chile; ^10^ Departamento de Ciencias, Facultad de Artes Liberales Universidad Adolfo Ibañez Santiago Chile

**Keywords:** Chile, heart rate, intertidal, metabolic rate, Mollusca, physiological performance

## Abstract

Biogeographic breaks represent crucial ecological junctures where species encounter novel environments that challenge their physiological limits and influence evolutionary trajectories. Two biogeographic breaks delineate distinct environmental regimes along the Chilean coastline on the Southeastern Pacific. In particular, the equatorward break, situated around 30° S–32° S, marks a transition from semi‐permanent upwelling to seasonal and intermittent upwelling poleward. The environmental break maintains a heterogeneous landscape for all coastal species along the region. Marine invertebrates, particularly intertidal ectothermic species such as limpets of the *Scurria* genus, exhibit diverse physiological responses to variation in the thermal environment. We characterized the physiological performance of five *Scurria* limpet species by measuring metabolic rate (oxygen consumption) and heart rate, together with buoyant weight as a proxy of shell calcification, under a standardized acclimation design under controlled laboratory conditions in individuals sourced from four locations spanning 17° of latitude, including the 30° S–32° S biogeographic break. We tested if the observed geographic variation in phenotypic traits was associated with differences in sea surface temperature, geographic location, and population genetic structure. Our findings indicate that the observed variation in metabolic traits among localities does not follow a latitudinal trend or mirror the biogeographic origin of the populations. We found no evidence supporting the role of latitudinal metabolic compensation or local adaptation in metabolism in explaining the distribution of limpets. However, significant differences in these traits among locations were observed, varying among species between sites but showing little to no correlation with the documented genetic structure.

## Graphical abstract

This study explores the geographic variation of different phenotypic traits in five *Scurria* limpet species along the Southeastern Pacific, revealing some significant trait–locality relationships but no correlation with genetic structure.

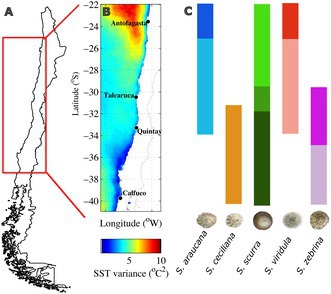

## 1 Introduction

Biogeographic breaks are areas where species experience novel evolutionary ecological interactions under environmental conditions that challenge their physiological limits (Kawecki 2008; Sexton et al. 2009). The shoreline of Chile, from 8° to 54° S, has been extensively utilized as a study system to understand the ecological and evolutionary dynamics of marine organisms over biogeographical scales (Gaitán‐Espitia et al. 2014; Gaitán‐Espitia, Bacigalupe, et al. 2017; Gaitán‐Espitia, Marshall, et al. 2017). Indeed, two biogeographic breaks are widely recognized along the Chilean shore (Camus 2001). An equatorward (northern) break, around 30° S–32° S, is marked by a major headland where a spatial discontinuity in the coastal upwelling regime takes place: weak semi‐permanent upwelling equatorward of the break and seasonal and intermittent poleward of it (Hormazabal et al. 2004; Thiel et al. 2007). The poleward (southern) break, around 39°–40° S, sits at the beginning of the Fiordland region of southern Chile (Camus 2001; Montecino and Lange 2009; Silva et al. 2009; Thiel et al. 2007). The northern and southern biogeographic breaks define three biogeographic provinces: (1) a Northern (Peruvian) province (from 4° to 30° S), (2) the Southern (Magellanic) province (42° S to 54° S), and (3) a transitional area between both provinces (from 30° S to 42° S) (Spalding et al. 2007). Following the different upwelling regimes around 30° S–32° S, the environmental transition signaled by the biogeographic break is primarily between different thermal regimes (Lara et al. 2019). Large‐scale spatial heterogeneity in ambient temperature has been shown to be an important determinant of ectotherm species distribution (Stuart‐Smith et al. 2017). Over large environmental filters, local adaptation to different environmental regimes can lead to differences in physiological plasticity among populations of broadly distributed invertebrate species (Gaitán‐Espitia et al. 2014; Lardies et al. 2021; Lardies et al. 2011; Pörtner et al. 2001). Unfortunately, few investigations have considered the influence of spatiotemporal environmental variation as a driver of geographic differences in phenotypic plasticity and genetic differentiation among populations of marine invertebrates (e.g., Broitman et al. 2018; Gaitán‐Espitia, Bacigalupe, et al. 2017; Gaitán‐Espitia, Marshall, et al. 2017; Dupont and Pörtner 2013; Lardies et al. 2014; Rivest et al. 2017). Marine invertebrates are ectotherms, which means that environmental temperature is the main abiotic factor affecting many of their organismal processes, biogeographic distributions, and habitat preferences (Sunday et al. 2011). Variation in environmental temperature is thus expected to produce physiological differences that can eventually result in local adaptation to different environmental regimes (Bozinovic et al. 2011; Watanabe and Payne 2023).

The limpets of the *Scurria* genus are broadly distributed along the rocky shores of the Pacific coast of South America, from northern Perú to southern Patagonia, where they occupy the mid‐to‐high intertidal zone levels (Aguilera et al. 2013); they are aragonite calcifiers. While the geographic range of some species spans the biogeographical zones highlighted above, others find the edge of their ranges in one of these breaks (Espoz et al. 2004; Lardies et al. 2021). As such, along with their geographical distribution, different populations of multiple *Scurria* species are exposed to contrasting environmental regimes (Broitman et al. 2021; Lara et al. 2019; Vargas et al. 2017). Standard metabolic rate (SMR) is a key bioenergetic trait for ectotherms that represents how an organism functions, integrating all simultaneous biochemical reactions and informing about the criterion of animal energy allocation (Bartheld et al. 2015; Nespolo et al. 2003). SMR is a repeatable and heritable trait (Bruning et al. 2013; Nespolo and Franco 2007); it correlates with changes in life histories (Lardies and Bozinovic 2006), it is the target of natural selection (Artacho and Nespolo 2009), and it shows adaptive differentiation across populations of the land snail *Cornu aspersum* (Gaitán‐Espitia and Nespolo 2014). For the *Scurria* species, previous work in two parapatric species revealed that these traits differ between species at their overlap area within the 30° S biogeographic break (Broitman et al. 2018). However, it remains unsolved whether latitudinal physiological variation is the product of local adaptation to different environmental conditions and whether it correlates with genetic differentiation of geographically separated populations.

In the present study, we characterized the patterns of environmental variability in the coastal ocean at four study sites across 17° of latitude and encompassing the vicinity of the two biogeographic breaks associated with the transitional and the Peruvian biogeographic regions. Using five species of *Scurria* limpets, we examined the effect of latitude, local patterns of environmental variability, and population‐level genetic differentiation on the magnitude of MR variation among populations. Using this information, we tested the hypothesis of whether phenotypic variation covaried with species' population genetic structure and different geographic locations along the Chilean coast, a proxy of contrasting thermal regimes associated with the biogeographic breaks along the Chilean coast.

## 2 Materials and Methods

### 2.1 Study Sites, Animal Collection, and Maintenance

Limpets of the species *Scurria zebrina, Scurria viridula, Scurria araucana, Scurria ceciliana*, and *Scurria scurra* were collected in four different sites along the coast of Chile, depending on their geographic distribution (see Table 1). Sampling was performed under permit R Ex. N 2036, 2019 (SUBPESCA) of the Chilean government. These sites were characterized by different patterns of variability in Sea Surface Temperature (SST), food supply, and upwelling dynamics (Aravena et al. 2014; Broitman et al. 2018; Lara et al. 2019; Torres et al. 2011; Vargas et al. 2017).

**TABLE 1:** Details of each of the study sites, including their geographic coordinates (Lat, Lon), the satellite‐derived statistics (average, variance, minima, and maxima) for sea surface temperature (SST) at each study site, and the number of experimental individuals of *Scurria limpets* from each location. For each species, different letters in parentheses indicate different genetic groups inferred from SNP‐derived genomic data (see main text for details).

Site	Sea surface temperature						Number of samples per *Scurria* species				
	Lat	Lon	avg.	var.	min.	max.	*araucana*	*ceciliana*	*scurra*	*viridula*	*zebrina*
Antofagasta	−23.56	−70.41	18.4	0.3	17.6	18.9	3 (a)	—	6 (a)	20 (a)	—
Talcaruca	−30.48	−71.7	12.6	0.7	11.7	13.4	20 (b)	—	15 (b)	14 (b)	14 (a)
Quintay	−33.29	−71.67	12.2	0.06	12.0	12.6	13 (b)	9 (a)	16 (b)	14 (b)	20 (a)
Calfuco	−39.75	−73.4	11.3	0.2	10.6	11.6	—	29 (a)	13 (c)	—	17 (b)

Firstly, 10–20 individuals were collected from each species and population during low tide during the spring–summer season. These individuals were transported in coolers to the laboratory and then measured to determine the relationship between shell length and calcium carbonate content (Buoyant Weight). Subsequently, in the laboratory, they were maintained in aquaria with seawater and each individual was marked and identified with bee tags (Beeworks) glued to the rear zone of the shell. The seawater for the aquariums was obtained from Quintay (33°11′ S; 71°1′ W, Valparaíso Region), and the animals were maintained with a salinity of 33 ppt and 14°C ± 1°C (using a water bath SunSun) for 14 days of acclimation and fed ad libitum with *Ulva* spp. The length of the acclimation period was chosen following the temporal scales of variation in environmental variables (i.e., O_2_, pH, *p*CO_2_, and temperature) in the region, which have an average duration of 13–15 days during upwelling activation (Ramajo et al. 2019).

Since *Scurria* limpets are intertidal organisms, we measured oxygen consumption of limpets underwater as a proxy for underwater metabolic rate, and we measured the heart rate of limpets in emersion conditions as a proxy for thermal performance. Recent studies have suggested that thermal stress in intertidal ectotherms is primarily associated with elevated water temperatures rather than air temperatures (Seabra et al. 2016). Body temperature of mollusks, however, is affected mainly in emersion periods (Helmuth et al. 2006), and heart rate has been reported as temperature‐dependent. The tidal regime along the coast of central‐northern Chile is symmetrical and semi‐diurnal, implying that organisms on the lower shore experience full aerial conditions only during spring and neap tides, which take place either during the early morning or late afternoon at similar hours along the latitudinal gradient (Lardies et al. 2011). Hence, we made all oxygen consumption measurements under immersion conditions and all heart rate measurements under emersion conditions and did not attempt to replicate performance under the full tidal cycle (Pereira et al. 2025).

**FIGURE 1:**
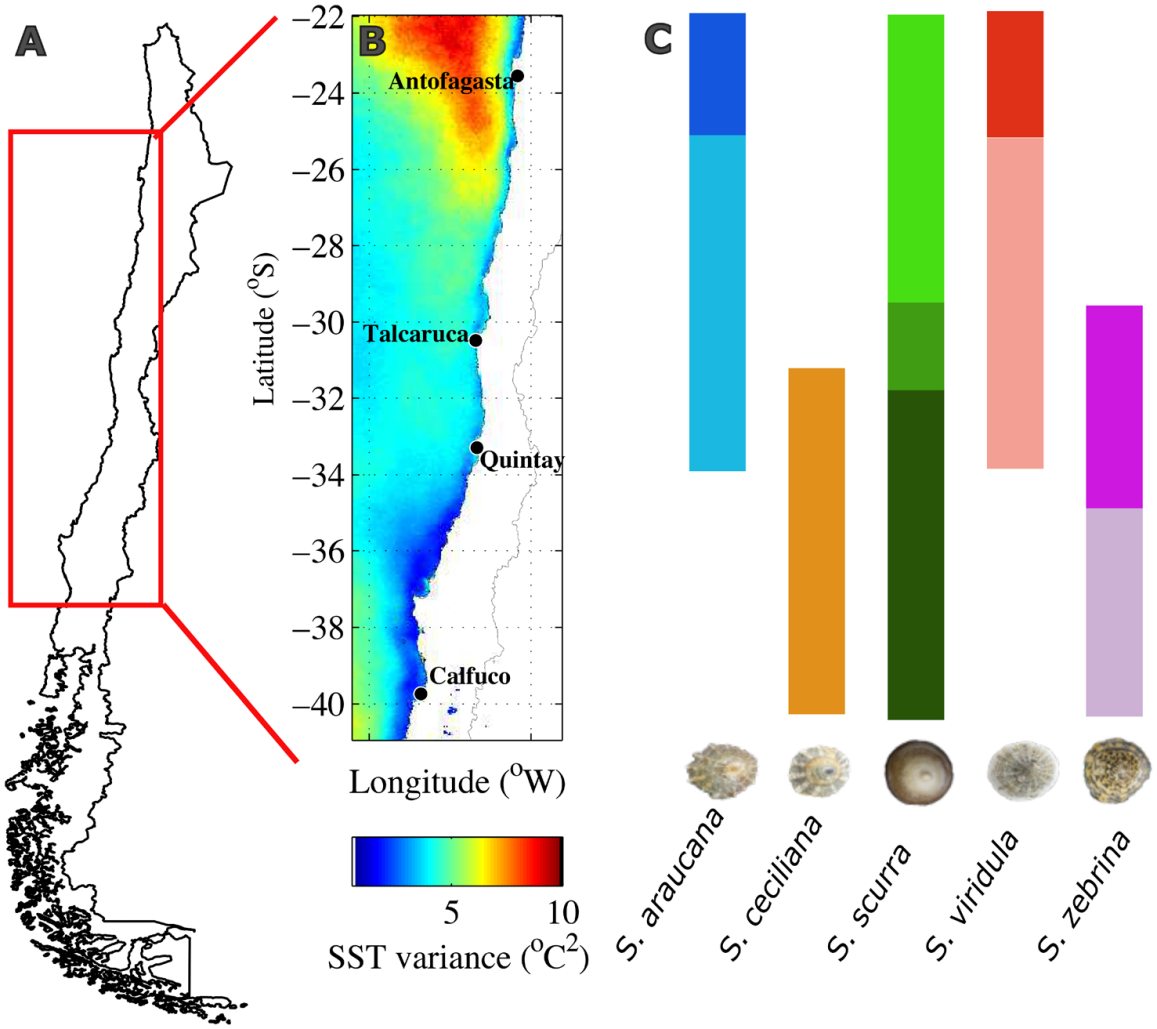
(A) Map of Chile indicating the study region (red rectangle). (B) Long‐term (2003–2018) SST variance (°C^2^) along the coast of our study region. The spatial pattern highlights the low variability in SST at the extremes of the study region and the regional maxima around Talcaruca. Notice that around Antofagasta variance is low along the coastal band while high variance offshore is driven by the interannual El Niño‐La Niña cycles. (C) Vertical bars indicate the geographic distribution of each species in the study area, in each bar, different color tonalities indicate different genetic groups as shown in Table 1 (for details on genetic data and methods see Peluso et al. 2023; Saenz‐Agudelo et al. 2022).

For metabolic and heart rate measurements, adult limpets were acclimated for 14 days at each of two experimental temperatures (14°C and 20°C ± 1°C). To avoid order effects, half of the individuals were first acclimated at 14°C and then transferred to 20°C, while the other half experienced the reverse sequence (20°C–14 C). The selection of these temperatures was based on a comprehensive monitoring program of intertidal temperature along the latitudinal gradient of the Chilean coast (Broitman et al. 2018; Gaitán‐Espitia et al. 2014). In this context, 14°C falls within the mean annual in situ Sea Surface Temperature (SST) range across all sites (Figure 1B), while 20°C represents a moderate to high increase from the mean annual SST, falling within the temperature extreme range experienced in all populations along the latitudinal gradient (Figure 1B,C). The 6°C increment in our experimental treatment aligns with the long‐term gradual warming trend of mean annual environmental temperature expected for the year 2100 under the business‐as‐usual scenario (RCP8.5) (Gattuso et al. 2015). All experimental procedures were performed following the guidelines of the bioethical committee of the Universidad Austral de Chile.

### 2.2 Environmental Characterization

Across the environmental gradient where the different populations and study sites were located, the aerial thermal regime at the shoreline is relatively stable. Conditions range from a hyperarid coastal desert (20° and 24° S) to a transitional zone between semi‐arid and Mediterranean regimes (30°–36° S) and a seasonal, temperate‐cold maritime regime (36°–41° S) (Di Castri and Hajek 1983). To quantify environmental conditions on the shoreline across the study region, we used satellite SST (°C) obtained from 8‐day night‐time level‐3 (L3) images at 4‐km resolution from the Moderate Resolution Imaging Spectroradiometer (MODIS). Using the L3 8‐day SST, we estimated environmental variability using different statistics of the temperature field around the study sites. We first extracted a 12 × 12 km square along and cross‐shelf from the location of the study site over four 8‐day images starting and prior to the 8‐day period when the animals were collected in the field for experiments. Using the spatiotemporal datasets, we calculated the mean temperature over 8, 16, and 32‐day periods, together with the variance, minima, and maxima over the 32‐day period, to integrate thermal variability over a time longer than the acclimation period (see below and Table 1).

### 2.3 Morphological and Metabolic Measurements

The calcium carbonate content of the limpets was estimated from buoyant weight (i.e., underwater weight) and verified with the dry weight measurements of the shells (Palmer 1992). Buoyant weight (*B*_w_) is a non‐invasive estimator of calcification rate and, thus, mollusk growth (Lardies et al. 2017; Palmer 1992). All the weight measurements were conducted using an analytical balance (Shimadzu AUX220). For different species of limpets, the calcium carbonate content and shell length were analyzed immediately after individuals arrived at the laboratory under controlled seawater parameters (see above).

Before oxygen uptake measurements, all individuals were kept under starvation for 24 h. Then, limpets were placed individually into a closed respirometric chamber (113 mL) filled with filtered seawater. Once sealed, the chambers were placed into a tank with seawater at 14°C controlled by a chiller. Oxygen consumption rates were measured using a fiber‐optic oxygen optode connected to a PreSens Microx TX3 temperature‐compensated oxygen meter (Precision Sensing, GmbH, Regensburg, Germany) with a tip diameter of 140 mm. Oxygen partial pressure measurements were run for at least 60 min and never allowed to decrease below 85% O_2_ saturation to avoid animals experiencing hypoxia. The first 10 min and the last 5 min of determinations were eliminated to avoid possible disturbances caused by the stress of animal manipulation. Furthermore, the same respirometric chambers, but without limpets inside, were used as controls (the control never had a reduction of the oxygen concentration higher than 3% of measurements). Each oxygen reduction due to background noise was subtracted from the individual measurements performed in the experimental chambers. Oxygen consumption values were expressed as mgO_2_ h^−1^ g^−1^. Each animal measured at 14°C was then acclimated by 14 days at 20°C or vice versa, and animals were randomly assigned to one of these temperature regimes, under the same feeding and photoperiod conditions (see Fernández et al. 2024), and SMR measurements were performed again.

### 2.4 Heart Rate Measurements

At the biogeographic transition zone around 30° S, characterized by fluctuating thermal conditions and low pH levels, marine benthic organisms face physiological challenges. In this study, we employed heart rate (cardiac activity) as a metric to assess how environmental and biogeographic factors influence the thermal performance of *Scurria* species. Following a two‐week acclimation period, 10–20 individuals per population were subjected to thermal sensitivity analysis. Each plastic chamber was subdivided into six sections, with one limpet placed in each section, and the chamber was immersed in a thermo‐regulated bath maintaining a constant seawater temperature (±0.5°C, LWB‐122D, LAB TECH) for 30 min. Experimental temperatures of 14°C and 20°C were selected to gauge thermal performance. Heart rate was measured using a heartbeat amplifier (AMP 03, Newshift Lda) and recorded using an oscilloscope (Handyscope HS4, TiePie Engineering, Sneek, The Netherlands). The frequency measures in Hertz (*f*_H_) signals were viewed and analyzed using TiePie Multi Channel software (version 1.0.29.0, TiePie Engineering). We recorded for 30 min, and the first and the last 5 min were discarded to avoid any noise or erroneous recordings generated by animal manipulation, and the results were expressed as the number of beats during 1 min (beats min^−1^). Heart rate cycles remained relatively stable throughout the 30‐min measurement period, even under high temperature, and we did not observe signs of fatigue or progressive decline in cardiac activity. Mean heart rate for each limpet at each experimental temperature was calculated to evaluate thermal plasticity within different populations. Heart rate was measured at the same period of the day to cancel the effects of a possible circadian or tidal rhythm of respiration.

### 2.5 Genetic Diversity

We used previously published genetic data (Peluso et al. 2023; Saenz‐Agudelo et al. 2022) to classify each of the populations sampled for each species into genetically differentiated groups. These genetic groups were inferred from genome‐derived SNP data. Briefly, we collected tissue samples from 10 to 12 individuals of each species in these locations, extracted genomic DNA, and characterized thousands of SNPs using the RADseq protocol (Baird et al. 2008). SNP matrices for each species were quality filtered and then used to evaluate genetic structure (for each species separately). The genetic cluster identity was then used as a categorical variable for further statistical analyses. Details on the genetic procedures and analyses can be found in the corresponding references (Peluso et al. 2023; Saenz‐Agudelo et al. 2022). Unfortunately, we could not obtain genetic data for the individual samples analyzed in this study, and the previous genetic studies did not include samples from Quintay. The allocation of a genetic group for Quintay was based on unpublished genetic data. Due to these limitations, we utilized the available genetic data to define genetic groups and explore potential correlations with the measured phenotypic traits. For reference, we report in the results the percentage of genetic variation explained by differences among these groups estimated in our previous work. These values were estimated in R using the package poppr (Kamvar et al. 2014).

### 2.6 Statistical Analyses

#### 2.6.1 Carbonate Content

Buoyant weight (*B*_w_) was analyzed in relation to shell length using a linear modeling framework. To assess whether the relationship between buoyant weight and shell length differed among localities, we modeled log‐transformed *B*_w_ as a function of log‐transformed shell length and sample locality, including interaction terms for each species.

#### 2.6.2 Metabolic Rate

Because *B*_w_ spanned two orders of magnitude, we used individual oxygen consumption data (VO_2_) per individual (mgO_2_ h^−1^) instead of per gram (mgO_2_ h^−1^ g^−1^), as previous studies have indicated that the use of mass‐specific units (i.e., VO_2_ per gram) is not recommended in this case (Hayes and Shonkwiler 1996). Individual oxygen consumption data (VO_2_) per individual (mgO_2_ h^−1^) was analyzed in relation to buoyant weight (*B*_w_) using a linear mixed effects modeling approach. To assess whether the relationship between metabolic rate and body mass differed across environmental conditions, we modeled log‐transformed VO_2_ per individual as a function of log‐transformed B_w_, temperature (14°C vs. 22°C), and sampling locality, including interaction terms. This approach allowed us to test whether both the slope and intercept of the Vo_2_‐*B*_w_ relationship varied across temperature and locality. To account for repeated measures (each individual was measured at two temperatures), we included sample ID as a random effect (Menzies et al. 2020; Nespolo et al. 2021). Separate models were fitted for each species using the lmer function from the lme4 R package (Bates et al. 2015). We repeated the same modeling approach, replacing the factor sampling locality with genetic group. This allowed us to test whether the slope and intercept of the VO_2_‐*B*_w_ relationship varied across temperature and genetic groups.

#### 2.6.3 Heart Rate

We examined whether the average heart rate (beats per minute) varied among localities or temperatures using a similar linear mixed effects modeling approach as for metabolic rate. For consistency, weight was also included as a variable in the full models. Sample ID was included as a random effect. We also repeated the modeling approach, replacing the factor sampling locality with the genetic group, to test whether both the slope and intercept of the VO_2_‐*B*_w_ relationship varied across temperature and genetic groups.

Following general linear model fitting (metabolic rate and heart rate), we conducted pairwise contrast analyses using estimated marginal means (EMMs) (Lenth 2025) to identify specific differences between localities within each temperature treatment and between temperatures within each locality. These targeted comparisons allowed us to test for biologically meaningful differences even in cases where the overall interaction term was not statistically significant.

For all models, homoscedasticity and normality of residuals were verified by visual inspection by plotting the residual and fitted values, as well as *Q–Q* plots of residuals.

## 3 Results

### 3.1 Environmental Characterization

Following the extensive latitudinal gradient included in our experimental design, we observed an important spatial gradient in SST. The largest difference in mean temperatures, over 8°C, was observed between Antofagasta and Calfuco, located at the extremes of our study region. The site located at the northern (equatorward) biogeographic break (Talcaruca) had the largest time‐integrated variance and the second‐lowest time‐integrated minimal temperature after the coldest site (Calfuco), adjacent to the southern (poleward) biogeographic break. The warmest site (Antofagasta) and the coldest site (Calfuco) showed similar patterns of time‐integrated thermal variability prior to the collection of the individuals used for the study (Table 1 and Figure 1).

### 3.2 Genetic Groups

The number of genetic groups identified from SNP data varied from a single genetic group for *S. ceciliana* to three genetic groups for *S. scurra*. Two genetic groups were identified for each of the remaining three species ( *S. araucana* , *S. viridula* , and *S. zebrina* ) (Table 1). The percentage of genetic variation explained by differences among groups differed among species. For *S. araucana* , differences among groups accounted for 3.17% of the total genetic variation; for *S. ceciliana*, only one group was found, and differences among localities accounted for 0.99% of the total genetic variation. For *S*. *scurra*, differences among groups explained 11.81% of total genetic variation. For *S. viridula* and *S. zebrina* , differences among groups explained 6% and 5.7% of total genetic variation, respectively.

### 3.3 Carbonate Content and Phenotypic Plasticity in Metabolic Rates

**FIGURE 2:**
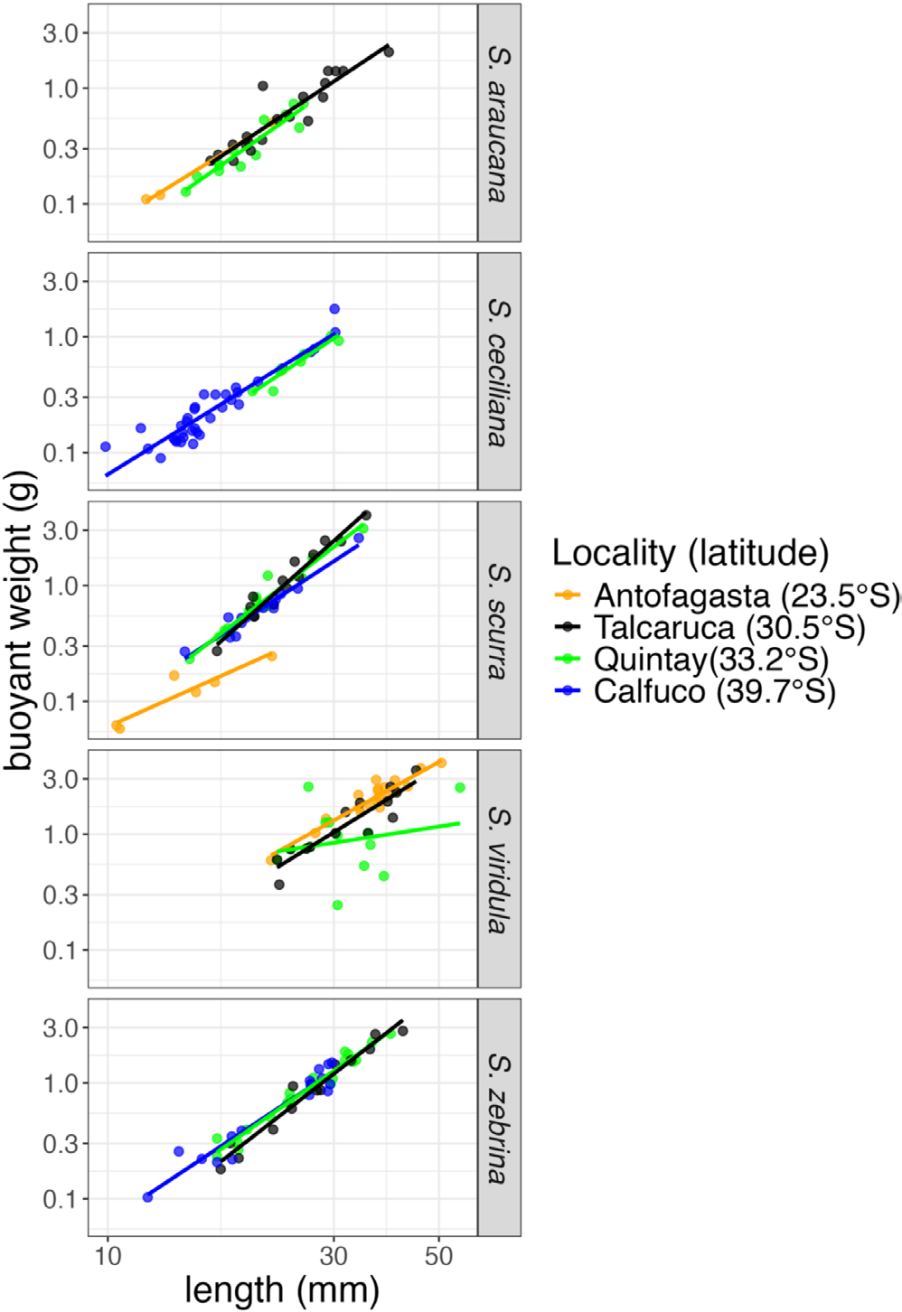
The length–buoyant weight relationship for each of the five *Scurria* species. Both axes are in log scale.

Among the five *Scurria* species, three of them ( *S. araucana* , *S. ceciliana*, and *S. zebrina* ) showed no variation among localities in the scaling relationship between buoyant weight and length. In contrast, for *S. scurra* and *S. viridula* , the scaling relationship was significantly different among localities (Figure 2, Table 2). For *S. viridula* , only the Quintay locality showed a scaling relationship significantly different from the other two (Antofagasta and Talcaruca) and showed higher variability and a shallower slope in this scaling relationship (Figure 2). In other words, *S. viridula* individuals from Quintay had less carbonate content in their shells than at the other localities. For *S. scurra*, individuals from Antofagasta displayed a smaller intercept and a gentler slope in the scaling relationship between buoyant weight and length; Talcaruca and Quintay showed the steepest slopes (Figure 2).

**TABLE 2:** Summary of the linear models testing for the allometric relationship between buoyant weight and length for each *Scurria* species. For each model, the locality was included as a cofactor.

Species	Variable	df	*F*	*p*
*S. araucana*	Length	1	289.78	< 0.001
	Locality	2	1.92	0.165
	Interaction	2	0.14	0.871
*S. ceciliana*	Length	1	384.85	< 0.001
	Locality	1	1.34	0.254
	Interaction	1	0.09	0.771
*S. scurra*	Length	1	1264.81	< 0.001
	Locality	3	20.43	< 0.001
	Interaction	3	9.51	< 0.001
*S. viridula*	Length	1	71.05	< 0.001
	Locality	2	8.41	0.001
	Interaction	2	4.92	0.012
*S. zebrina*	Length	1	905.36	< 0.001
	Locality	2	2.00	0.147
	Interaction	2	1.76	0.184

### 3.4 Metabolic Rate

**FIGURE 3:**
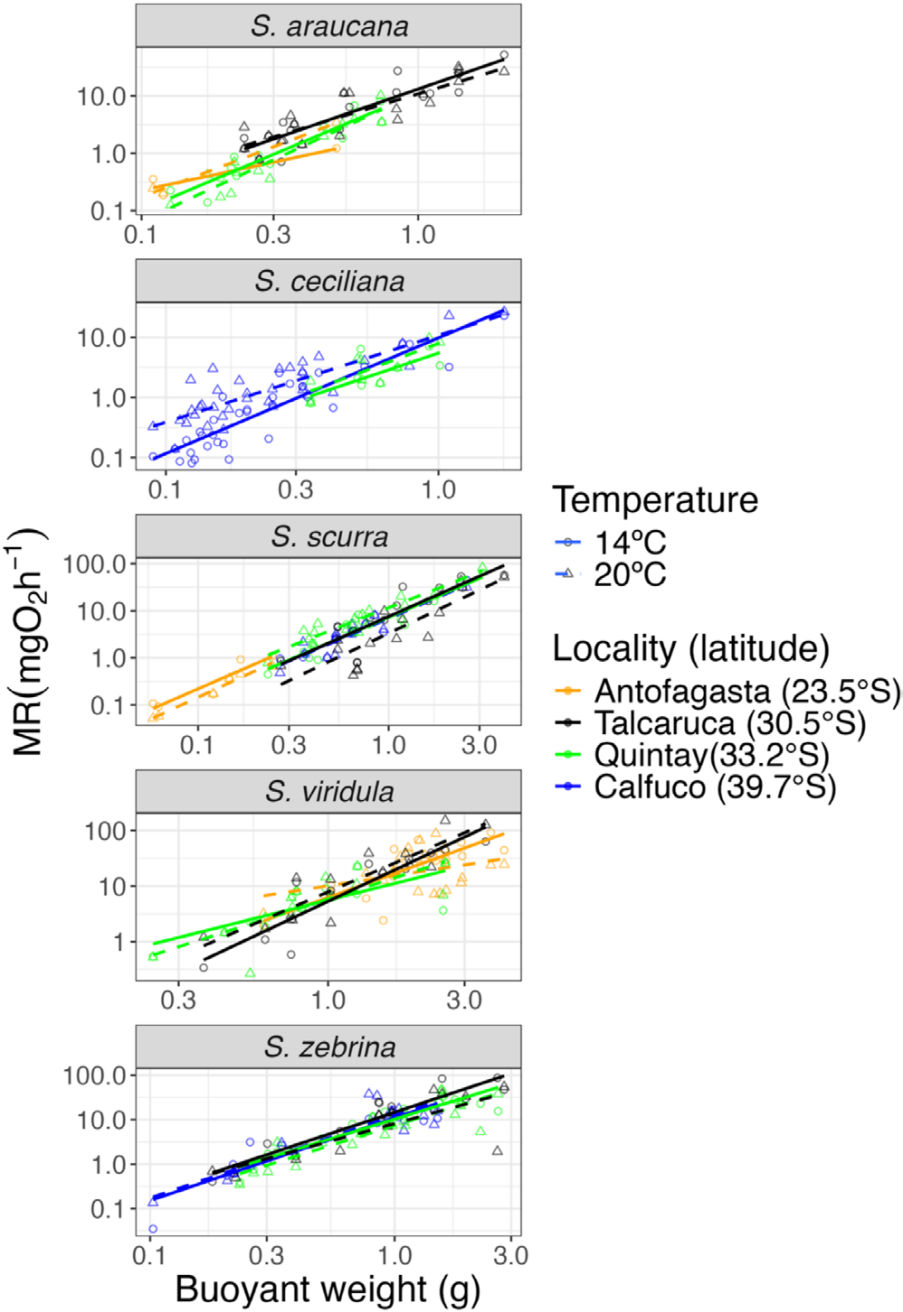
Metabolic rate—buoyant weight allometries for each *Scurria* species measured in different localities and under two experimental temperatures. Both axes are in log scale. Regression lines are fitted separately for each temperature and locality combination.

**FIGURE 4:**
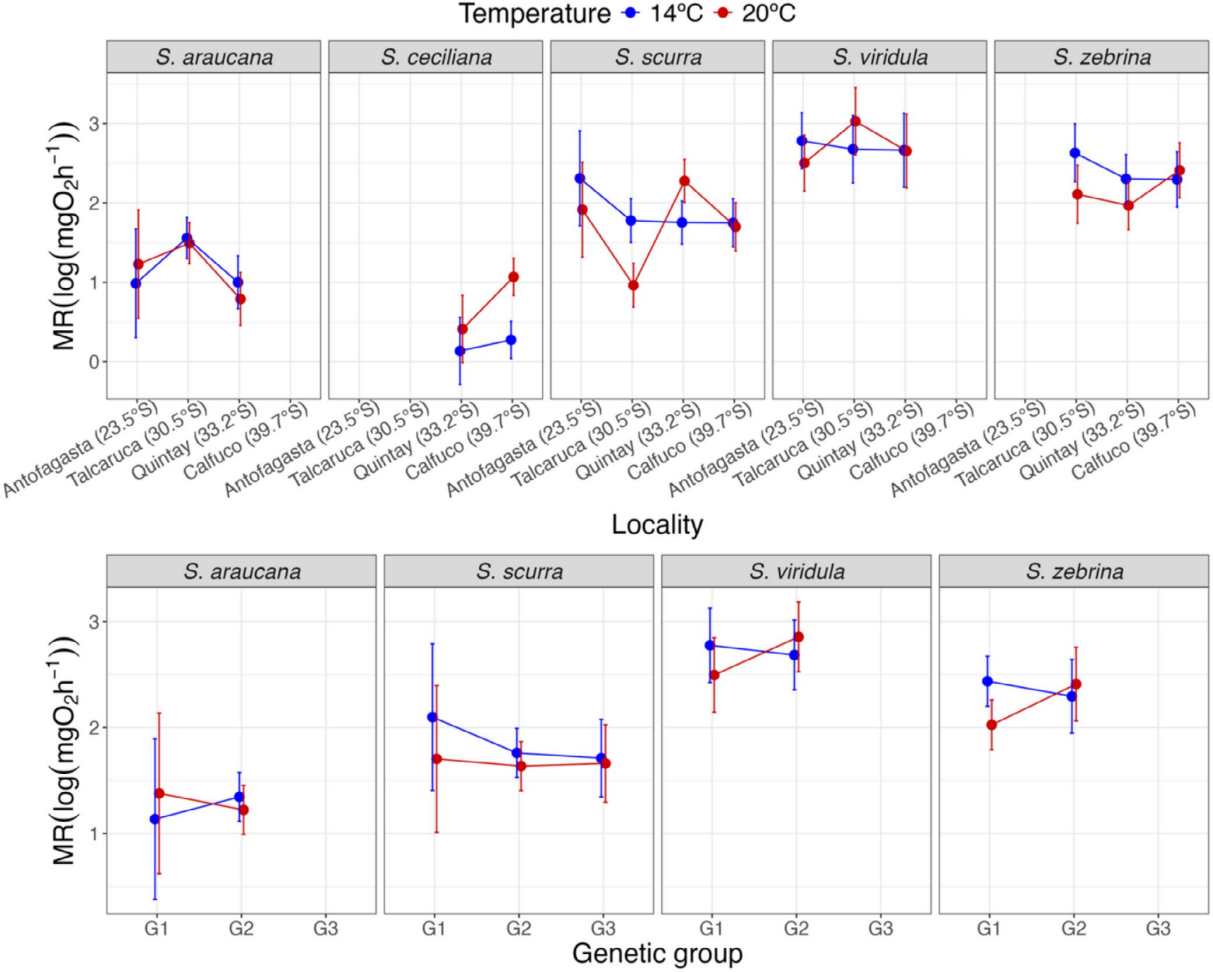
Estimated marginal means of metabolic rate (log_10_ mg O_2_ h^−1^) across localities (top panels) or genetic groups (bottom panels) and temperature treatments for each *Scurria* species. Points represent estimated marginal means from generalized linear models, with error bars showing 95% confidence intervals. Blue and red colors indicate different temperature treatments, and lines connect the same temperature treatment among localities (top panels) or genetic groups (bottom panels).

The results of generalized linear models (GLMs) showed that metabolic rate data log(mgO_2_h^−1^) displayed significant allometric correlation with buoyant weight (log scaled) (as a measurement of body mass) for all species (Table 3A, Figure 3). The slope of this correlation (exponent of the allometric equation) differed between temperature treatments for *S. ceciliana*, *S. scurra*, and *S. zebrina* and differed among localities for *S. araucana* , *S. scurra*, and *S*. *ceciliana* (Table 3A, Figure 3). There was a significant temperature–locality interaction only for *S. scurra* (Table 3A, Figure 3). Figure 4 (top panels) shows the EMMs of metabolic rates across localities and temperature treatments for each species derived from the GLMs. Temperature effects varied significantly among species and localities. *S. araucana* showed consistently higher metabolic rates at Talcaruca compared to Quintay at both temperatures (14°C: *p* = 0.028; 20°C: *p* = 0.004). *S. ceciliana* exhibited significantly higher metabolic rates at 20°C than at 14°C, but only at Calfuco (*p* < 0.001). *S. scurra* showed contrasting thermal responses across localities: significantly higher metabolic rates at 20°C than at 14°C at Quintay (*p* < 0.001), but significantly higher rates at 14°C than at 20°C at Talcaruca (*p* < 0.001), and displayed the most complex spatial pattern for the 20°C temperature, with significant differences among multiple localities (only the Antofagasta‐Calfuco and Antofagasta–Quintay contrasts were non‐significant, *p* > 0.05). *S. viridula* showed no significant temperature or locality effects. In *S. zebrina*, metabolic rate was significantly higher at 14°C than at 20°C at Talcaruca (*p* = 0.018).

**TABLE 3:** Linear mixed effects model results for all *Scurria* species showing Type III Analysis of Variance with Satterthwaite's method for denominator degrees of freedom. The response variable was log‐transformed metabolic rate, with log‐transformed buoyant weight as a covariate, temperature and (A) location as categorical fixed effects and their two‐way interaction; (B) genetic group and temperature as categorical effects and their two‐way interaction.

Species	Factor	Numdf	Dendf	*F*	*p*
**A. Type III analysis of variance table for locality and temperature**					
*S. araucana*	log(body weight)	1	39.3	231.47	< 0.001
	Temperature	1	40.9	0.01	0.936
	Location	2	35.4	7.08	0.003
	Interaction	2	40.9	0.73	0.486
*S. ceciliana*	log(body weight)	1	88	271.18	< 0.001
	Temperature	1	88	11.17	0.001
	Location	1	88	4.67	0.033
	Interaction	1	88	2.62	0.109
*S. scurra*	log(Body weight)	1	50	332.39	< 0.001
	Temperature	1	50	4.44	0.04
	Location	3	50	5.82	0.002
	Interaction	3	50	14.62	< 0.001
*S. viridula*	log(body weight)	1	48	118.43	< 0.001
	Temperature	1	48	0.03	0.873
	Location	2	48	0.55	0.58
	Interaction	2	48	2.12	0.131
*S. zebrina*	log(body weight)	1	51	338.11	< 0.001
	Temperature	1	51	5.03	0.029
	Location	2	51	1.07	0.352
	Interaction	2	51	2.83	0.068
**B. Type III analysis of variance table for genetic group and temperature**					
*S. araucana*	log(body weight)	1	39.8	230.12	< 0.001
	Temperature	1	40.9	0.11	0.747
	Genetic group	1	354	0.01	0.934
	Interaction	1	40.9	0.97	0.33
*S. scurra*	log(body weight)	1	50	236.9	< 0.001
	Temperature	1	50	1.94	0.17
	Genetic group	2	50	0.26	0.769
	Interaction	2	50	0.41	0.664
*S. viridula*	log(body weight)	1	48	128.32	< 0.001
	Temperature	1	48	0.17	0.678
	Genetic group	1	48	0.42	0.521
	Interaction	1	48	2.99	0.09
*S. zebrina*	log(body weight)	1	51	327.25	< 0.001
	Temperature	1	51	1.63	0.207
	Genetic group	1	51	0.53	0.47
	Interaction	1	51	5.15	0.027

Abbreviations: Dendf, denominator degrees of freedom; Numdf, numerator degrees of freedom.

Temperature effects varied among genetic groups within species, though most comparisons were non‐significant. Only *S. zebrina* exhibited a significant genetic group‐specific thermal response: genetic group 1 showed significantly higher metabolic rates at 14°C than at 20°C (*p* = 0.004), while genetic group 2 showed no significant temperature effect (*p* = 0.554). Additionally, *S. zebrina* showed a marginally significant difference between genetic groups at 20°C, with genetic group one displaying a lower metabolic rate than group 2 (*p* = 0.068) (Figure 4, bottom panels).

### 3.5 Heart Rate

**FIGURE 5:**
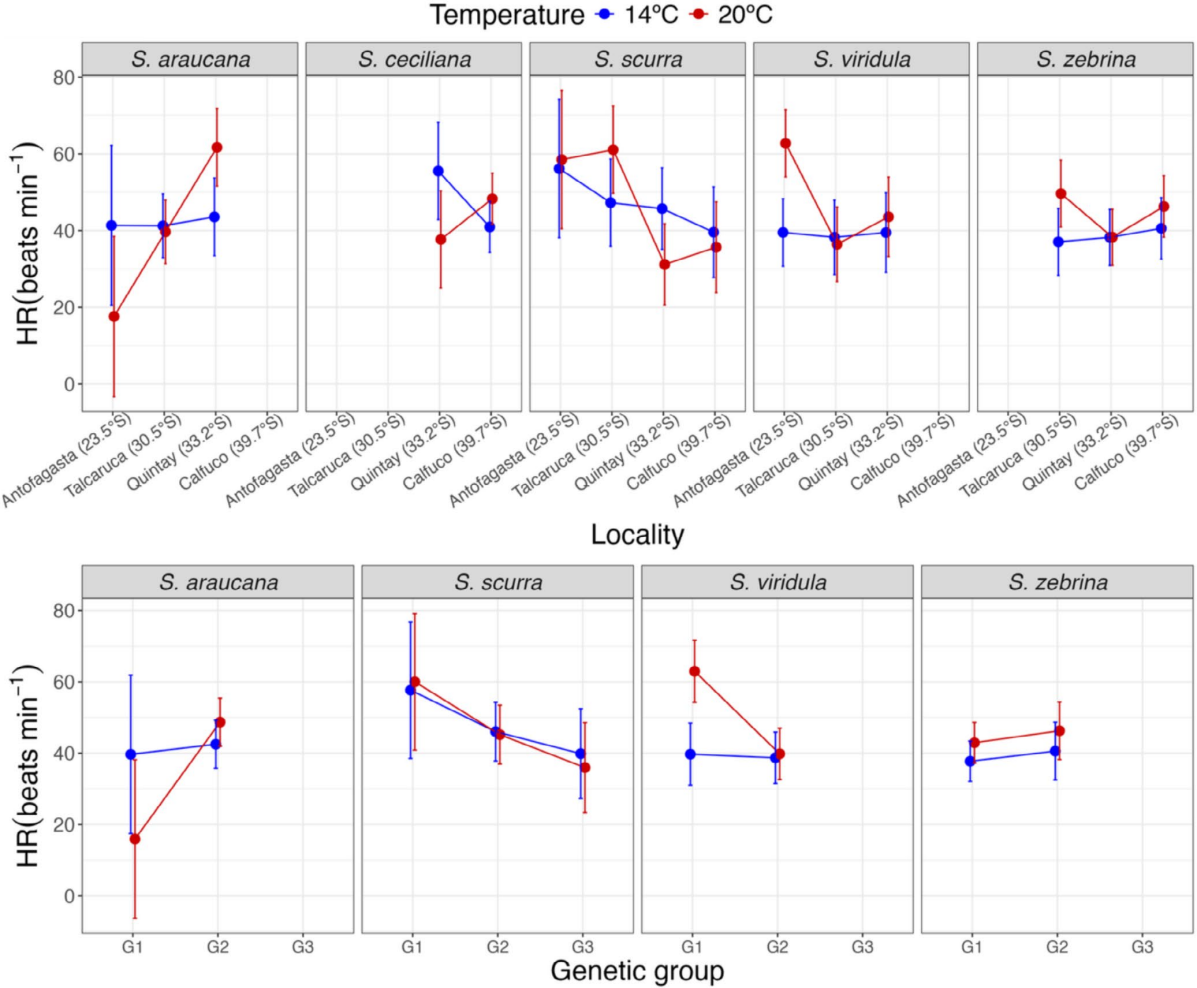
Estimated marginal means of heart rate across localities, genetic groups and temperature treatments for each *Scurria* species. Points represent estimated marginal means from generalized linear models, with error bars showing 95% confidence intervals. Blue and red colors indicate different temperature treatments, and lines connect the same temperature treatment among localities (top panels) or genetic groups (bottom panels). *S. ceciliana* was excluded from the bottom panel.

The results of the GLMs showed that body weight was not significantly correlated with heart rate for any of the species. A summary of the modeling results is found in Table 4A. For *S. araucana* and *S. scurra*, the models showed a significant effect of locality and an interaction between locality and temperature. For *S. ceciliana*, neither temperature nor locality had a significant effect, but there was a significant interaction between them (Table 4A). For *S. viridula* and *S. zebrina* , the model indicated a significant effect of temperature but not locality and an interaction between temperature and locality. Figure 5 (top panels) depicts the EMMs for heart rate measurements for different localities or genetic groups and temperature treatments. In general, heart rate showed significant differences among localities, mostly when measured at 20°C. For *S. araucana* , mean heart rate at 20°C was significantly lower at Antofagasta compared to Quintay (*p* < 0.001) and lower at Talcaruca compared to Quintay (*p* < 0.001). In contrast, for *S. scurra* and *S. viridula* , heart rate at 20°C was significantly higher in northern locations compared to southern ones (*S. scurra*: Antofagasta—Quintay, *p* < 0.001; Talcaruca—Quintay, *p* = 0.001; Talcaruca—Calfuco, *p* = 0.015; *S. viridula* : Antofagasta—Talcaruca, *p* < 0.001) (Figure 5). There were no significant differences among locations for any of the heart rate means at either temperature for *S. zebrina* . Temperature effects on mean heart rate varied significantly among locations within species. In *S. araucana*, heart rate was significantly lower at 14°C than at 20°C only at Quintay (*p* = 0.011), while other locations showed no significant temperature response (Figure 5). *S. ceciliana* exhibited the opposite pattern at Quintay, with significantly higher HR at 14°C (*p* = 0.030). *S. scurra* showed the most variable responses, with significantly higher rates at 14°C in Quintay (*p* = 0.023) but significantly lower rates at 14°C in Talcaruca (*p* = 0.037). *S. viridula* also showed a temperature response, with higher heart rates at 20°C in Antofagasta (*p* < 0.001), but no significant temperature effects at other locations. Finally, *S. zebrina* showed significant temperature differences only at Talcaruca, with lower rates at 14°C (*p* = 0.007) (Figure 5, top panels).

**TABLE 4:** Linear mixed effects model results for all species showing Type III Analysis of Variance with Satterthwaite's method for denominator degrees of freedom. The response variable was heart rate, with buoyant weight as a covariate, temperature and (A) location as categorical fixed effects and their two‐way interaction; (B) genetic group and temperature as categorical effects and their two‐way interaction.

Species	Factor	Numdf	Dendf	*F*	*p*
**A. Type III analysis of variance table for locality and temperature**					
*S. araucana*	Body weight	1	37.1	0.95	0.337
	Temperature	1	45.5	0.2	0.658
	Location	2	41.7	6.04	0.005
	Interaction	2	45.5	4.9	0.012
*S. ceciliana*	Body weight	1	83.9	0.04	0.85
	Temperature	1	53.9	1.42	0.238
	Location	1	40.4	0.13	0.72
	Interaction	1	53.9	8.2	0.006
*S. scurra*	Body weight	1	50	1.11	0.297
	Temperature	1	50	0.03	0.874
	Location	3	50	4.36	0.008
	Interaction	3	50	3.73	0.017
*S. viridula*	Body weight	1	48	1.04	0.312
	Temperature	1	48	24.49	< 0.001
	Location	2	48	2.62	0.083
	Interaction	2	48	21.63	< 0.001
*S. zebrina*	Body weight	1	51	1.79	0.187
	Temperature	1	51	6.93	0.011
	Location	2	51	0.88	0.423
	Interaction	2	51	2.42	0.099
**B. Type III analysis of variance table for genetic group and temperature**					
*S. araucana*	Body weight	1	39.9	3.85	0.057
	Temperature	1	46.9	1.37	0.247
	Genetic group	1	44.7	4.52	0.039
	Interaction	1	46.9	3.99	0.052
*S. scurra*	Body weight	1	50	2.69	0.107
	Temperature	1	50	0.03	0.863
	Genetic group	2	50	2.85	0.067
	Interaction	2	50	0.13	0.883
*S. viridula*	Body weight	1	48	0.81	0.371
	Temperature	1	48	48.55	< 0.001
	Genetic group	1	48	4.75	0.034
	Interaction	1	48	40	< 0.001
*S. zebrina*	Body weight	1	51	1.83	0.182
	Temperature	1	51	4.58	0.037
	Genetic group	1	51	0.56	0.46
	Interaction	1	51	0.01	0.923

Abbreviations: Dendf, denominator degrees of freedom; Numdf, numerator degrees of freedom.

When genetic groups were included in the models, a significant effect of temperature was only observed for *S. viridula* and *S. zebrina* , a significant effect of genetic group was observed for *S. araucana* and *S. viridula* , and a significant interaction between genetic group and temperature was found only for *S. viridula* (Table 4B). Figure 5 (bottom panels) illustrates the marginal mean heart rates per locality and genetic group measured at the two different temperatures for each species. Significant differences in marginal means in heart rate among localities were only observed when measured at 20°C for *S. araucana* (genetic group 1 lower than genetic group 2, *p* = 0.006) and *S. viridula* (genetic group 2 lower than genetic group 1, *p* < 0.001) (Figure 5). Within genetic groups, significant differences between temperature treatments were only found in *S. viridula* for G1 (HR at 20°C higher than at 14°C, *p* < 0.001).

## 4 Discussion

Our study investigated the variation of three physiological traits (metabolic rate, heart rate, and carbonate content) of five species of *Scurria* limpets across a latitudinal gradient spanning 17 degrees of latitude and whether phenotypic variation covaried with species' population genetic structure and sampling locality, a proxy of environmental heterogeneity. Overall, our results indicate that, despite the substantial environmental gradient (over 8°C difference in average sea temperature between extreme localities), physiological responses to temperature were highly context‐dependent, varying among species and localities rather than following a simple latitudinal pattern. Broadly, these results appear to rule out that a simple latitudinal metabolic compensation can serve as a primary explanation for the observed distribution of these limpets. However, our results indicate significant differences in physiological traits among locations that varied among species: metabolic rate showed locality effects in three of the five species (*S. araucana, S. ceciliana*, and *S. scurra*), heart rate differed among localities in four species ( *S. araucana* , *S. scurra*, *S. viridula* , and *S. zebrina* ), and carbonate content scaling relationships varied among localities in two species (*S. scurra* and *S. viridula* ). Genetic groups had minimal and inconsistent effects on both metabolic and cardiac traits, with most genetic group comparisons being non‐significant. Below, we discuss these results in terms of five key ideas.

Several authors proposed that metabolic performance could determine distribution limits in marine invertebrates, particularly for limpets (Deutsch et al. 2015; Broitman et al. 2018; Virgin and Schiel 2023). Recent evidence indicates that marine gastropods are largely insensitive to temperature variations due to their high capacity for respiratory compensation (Marshall and McQuaid 2020). Indeed, limpets have a particularly high upper thermal tolerance under stressful conditions (Bjelde et al. 2015), which would explain the lack of change in MR across the large latitudinal gradient examined in our study. In addition, the dispersive larval phase of *Scurria* limpets points to the structure of ocean currents and geographic breaks as major drivers of their geographic distribution. A recent study that compared the observed genetic structure for *S. scurra* and *S. araucana* and patterns of population connectivity inferred from biophysical simulations of larval dispersal indicates that the oceanographic setting is a good predictor of the observed genetic breaks (Peluso et al. 2024). While it seems unlikely that adult metabolic performance is a driver of local adaptation, environmental filters during the larval stage may be in part responsible for the spatial patterns of genetic structure. However, the local importance of metabolic rate remains to be tested in an experimental setting. On the other hand, both body size and metabolic rate in the intertidal mussel *Perumytilus purpuratus* show a strong and direct dependence on latitude and sea surface temperature along the Southeastern Pacific, and they also respond to multiple environmental drivers such as *p*CO_2_, alkalinity, salinity, and pH (Labra et al. 2022). This example highlights the importance of assessing the effects of additional environmental drivers on both phenotypic traits of our model species.

Our results show little variation in calcification rates (buoyant weight) among localities for all species. Only *S. scurra* and *S. viridula* displayed significant variation, and only *S. viridula* displayed a reduction in the slope and higher dispersion in the *B*_W_‐length correlation at a single locality, Quintay. Our analysis accounted for variation in the buoyant weight–length relationship through log–log scaling and population‐level interactions. Nonetheless, differences in shell shape can also affect this relationship (Trussell 2000). Future work using explicit morphometric descriptors would help clarify how shape influences carbonate content across populations. The Quintay location was colonized by *S. viridula* following this species' recent geographic range expansion that started less than 100 years ago (Aguilera et al. 2013; Rivadeneira et al. 2002). Previous studies that measured the physiological variation of *S. viridula* and *S. zebrina* around the 30° S biogeographic break (Broitman et al. 2018; Lardies et al. 2021) showed similar results. Calcification is a costly metabolic process, and the higher variation in calcification rates at Quintay is likely linked to the metabolic constraints that the environment imposes on this species at its southern range limit. For *S. scurra*, it is interesting to note that the difference relates to the northern locality displaying a lower intercept in the buoyant weight–length relationship, which matches both a genetic transition in the genetic structure of *S. scurra* (Peluso et al. 2023) as well as the biogeographic break of the distribution between its two cryptic macroalgal host species, *Lessonia spicata* poleward and *L. berterona* equatorward (Tellier et al. 2009).

In general, most of the variation in metabolic rate was explained by body mass (buoyant weight). However, there were significant responses in terms of metabolic rate when different species were exposed to 14 vs. 20°C. We found that for *S. scurra* metabolic rate had a drastic change between Talcaruca and Quintay when measured at 20°C. In particular, the higher‐than‐expected metabolic rate at Quintay suggests that 20°C is stressful for *S. scurra* in this locality. It is worth noting that Talcaruca and Quintay displayed contrasting differences in metabolic rate at 20°C despite both localities belonging to the same genetic group (Peluso et al. 2023). It is important to note that *S. scurra* is the only species in our study that is restricted to the lower part of the intertidal zone, where it homes onto stipes of *Lessonia* kelps, while the other species are restricted to the mid‐intertidal zone. Large El Niño events have been shown to extirpate *L. berteroana* along southern Perú and northern Chile (Castilla and Camus 1992). In this way, it is plausible that *S. scurra* populations located inside and around the 30°C biogeographic break display physiological responses differing from the populations from the other study locations (Broitman et al. 2018). This dependence of *S. scurra* on host algae could generate local selective pressures that modulate tolerance limits, potentially explaining why populations from Talcaruca and Quintay—although belonging to the same genetic group—displayed contrasting metabolic responses at 20°C. Under this perspective, the interaction between host distribution and climate variability emerges as a broader mechanism influencing physiological differentiation among populations. The notion of diversity in adaptation strategies around biogeographic breaks is an idea that remains to be addressed in future studies (Hampe and Petit 2005). Interestingly, for *S. zebrina* we also found a significant response in metabolic rate to different temperatures at Talcaruca and a significant interaction between temperature and genetic group. These results are consistent with previous genetic analyses that reported the presence of SNPs with deviations from neutral expectations in allele frequency distributions when comparing populations from these two genetic groups (Saenz‐Agudelo et al. 2022). We note that the small differences or marginal interaction between locality and temperature are likely the consequence of our small sample size and limited number of sampled localities. Despite these caveats, whether our results indicate local adaptation to different environments across this biogeographic break linked to heritable changes in metabolic rate requires further experimentation in a more rigorous framework.

Variation in heart rate was more important than variation in metabolic rate. There was a significant locality/temperature interaction for all but one species. Interestingly, this interaction was driven by important changes in heart rate for individuals measured at 20°C, while heart rate measured at 14°C remained relatively constant among localities for all species. The pattern of heart rate for *S. scurra* mirrors the pattern found for metabolic rate. *S*. *scurra* inhabits the lower part of the intertidal zone; hence, it seldom encounters fully aerial conditions (i.e., inhabits in normoxia) and probably cannot decouple heart rate from metabolic rate (Gurr et al. 2018; Marshall and McQuaid 2020). A significant interaction between genetic group and temperature was observed only for *S. viridula* , with marginally significant effects for *S. araucana*, though these interactions showed opposite patterns between species (northern genetic groups had lower heart rates at 20°C in *S. araucana* but higher heart rates at 20°C in *S. viridula*). While the limited genetic group–temperature interactions suggest that much of the observed physiological variation may reflect phenotypic plasticity, we cannot exclude local adaptation as a contributing factor. This is in part because the genetic groups used here are based on genome‐wide SNPs that primarily capture neutral genetic variation and may not adequately detect adaptive divergence at functionally relevant loci. Indeed, previous genomic studies that targeted a fraction of the genome (RADseq) have found evidence of adaptive divergence correlated with environmental differences for *S. araucana* (Peluso et al. 2024) but not for *S. viridula* (Saenz‐Agudelo et al. 2022). However, recent whole‐genome resequencing data of *S. viridula* showed similar patterns (Giles et al. 2025), supporting the idea of phenotypic plasticity of heart rate in *S. viridula* . That being said, we acknowledge that ultimately distinguishing between phenotypic plasticity and local adaptation will require common garden experiments. Our results nevertheless confirm that ectothermic organisms in temperate regions exhibit a high degree of phenotypic plasticity in response to environmental parameters such as temperature, producing reaction norms over an environmental filter (Broitman et al. 2021; Scheiner 1993).

At the intraspecific level, previous studies have documented decreases in metabolic rates among high‐latitude populations of vertebrate and invertebrate ectotherms (Angilletta 2001; Antiqueira et al. 2022; Barria et al. 2018; Chown et al. 1998; Labra et al. 2022; Osores et al. 2018). We controlled for confounding factors such as seasonality, life stage, and activity by timing the moment, size, and microenvironment of individual collection, respectively, while diet, experimental technique, and temperature acclimation period were controlled in the laboratory (Broitman et al. 2018). Specifically, within our range of experimental temperatures, we observed no pattern in either metabolic rate or heart rate with latitude. Consequently, our findings do not support the notion that a higher metabolic rate confers an advantage to limpets inhabiting cooler environments and underscore the well‐established relationship between metabolic rate, temperature, and body size in all ectothermic organisms (Clarke and Johnston 1999; Labra et al. 2022). Thus, our study provides direct evidence of both intraspecific and interspecific latitudinal disparities in metabolism. Moreover, we suggest that limpets in the biogeographic break would show greater signs of phenotypic plasticity, given the wide environmental variability they experience (Barria et al. 2014; Broitman et al. 2021; Naya et al. 2011).

Finally, our study shows that a biogeographic break, which is characterized by large environmental heterogeneity between sites over a narrow region, maintains a transition in genetic structure for multiple *Scurria* species (Broitman et al. 2018; Fernández et al. 2024). The spatial genetic structure did not directly translate to the species' physiological performance. However, we observed significant differences in performance in individuals sourced from the sites inside and around the transitional zone between (i) the species that live in the more constant environment (low intertidal zone) and (ii) the species that has recently expanded its range poleward and invaded the transitional zone. For (i) in particular, the match between the presence of a genetic cluster and differences in physiological responses is likely related to the change in the species of macroalgal host, suggesting an environment/genotype mismatch. In the case of (ii), between‐site differences in performance have been shown before (Broitman et al. 2018), in agreement with expectations for leading‐edge populations. Together, our results suggest that the biogeographic transition zone represents an area of particular interest for conserving potentially unique genetic entities and hence of special concern for managing the multiple exploited species that inhabit this region.

## Author Contributions

**Pablo Saenz‐Agudelo:** conceptualization (equal), data curation (equal), formal analysis (equal), funding acquisition (lead), investigation (equal), methodology (equal), project administration (lead), resources (equal), writing – original draft (equal), writing – review and editing (equal). **Roberto F. Nespolo:** conceptualization (equal), data curation (equal), formal analysis (equal), funding acquisition (supporting), investigation (equal), methodology (equal), project administration (supporting), writing – original draft (equal), writing – review and editing (equal). **Bernardo R. Broitman:** conceptualization (equal), data curation (equal), formal analysis (equal), funding acquisition (supporting), investigation (equal), methodology (equal), writing – original draft (equal), writing – review and editing (equal). **Paz Caballero:** formal analysis (supporting), investigation (supporting), methodology (supporting), writing – review and editing (supporting). **Marco A. Lardies:** conceptualization (equal), data curation (equal), formal analysis (equal), funding acquisition (equal), investigation (equal), methodology (equal), project administration (equal), resources (equal), writing – original draft (equal), writing – review and editing (equal).

## Conflicts of Interest

The authors declare no conflicts of interest.

## Supporting information


**Table S1:** Individual physiological measurements that were used in the article. For each individual, the following data are provided: Species ID, sampling site, genetic group, individual ID, shell length, shell width, shell height, buoyant weight, oxygen consumption per individual per hour at 14°C (mgO_2__h_g_14C) and at 20°C, (mgO_2__h_g_20C), and heart rate measured at 14°C and 20°C.

## Data Availability

All raw physiological measurements used in this study are available as Supporting Information (Table S1).
